# Phase contrast microscopy of living cells within the whole lens: spatial correlations and morphological dynamics

**Published:** 2012-08-01

**Authors:** Zhiying Kong, Xiangjia Zhu, Shenghai Zhang, Jihong Wu, Yi Luo

**Affiliations:** 1 Department of Ophthalmology, Eye & ENT Hospital, Fudan University, Shanghai, P.R. China; 2 Experimental Research Center, Eye & ENT Hospital, Fudan University, Shanghai, P.R. China

## Abstract

**Purpose:**

Images from cultured lens cells do not convey enough spatial information, and imaging of fixed lens specimens cannot reveal dynamic changes in the cells. As such, a real-time, convenient approach for monitoring label-free imaging of dynamic processes of living cells within the whole lens is urgently needed.

**Methods:**

Female Wistar rat lenses were kept in organ culture. Insulin-like growth factor-I was added to the culture medium to induce cell mitosis. A novel method of ultraviolet (UV) irradiation was used to induce cell apoptosis and fiber damage. The cellular morphological dynamics within the whole lens were monitored by inverted phase contrast microscopy. Apoptosis was assessed using a commercial kit with Hoechst 33342/YO-PRO®-1/propidium iodide (PI).

**Results:**

The intrinsic transparency and low-light scattering property of the rat lens permitted direct imaging of the lens epithelial cells (LECs) and the superficial fiber cells. We visualized the processes of mitosis and apoptosis of the LECs, and we obtained dynamic images of posterior fiber cells following UVA irradiation.

**Conclusions:**

This method opens a new window for observing lens cells in their physiologic location, and it can be readily applied in studies on lens physiology and pathology.

## Introduction

The transparency of the crystalline lens has been attributed to the complex, ordered arrangement of its components at both microscopic and molecular levels [1]. Morphological changes of the lens cells would result in light scattering and cataract. Despite tremendous advances in light microscopy, visualizing live-cell dynamics in dense, unstained specimens, such as the crystalline lens, has been problematic. Using fluorescence microscopy for tissue imaging (e.g., confocal microscopy) requires the use of dyes to visualize the cells; however, these dyes and lasers can be damaging to living cells [2-4]. Images from cultured cells do not convey enough spatial information and, once dissociated from their physiologic location, the cultured cells lose their intrinsic interactions with the environment [2]. Fixed histological section imaging cannot reveal dynamic changes in lens cells, and the lens loses its transparency once it is fixed. Therefore, a real-time, convenient approach for monitoring label-free imaging of cellular dynamics within the whole lens is urgently needed.

Current microscope technology for observing unstained living cells (such as phase contrast and differential interference contrast microscopy) is mainly based on the principle of light transmission and refraction [2,5,6]. Unfortunately, most biologic tissues are not suitable for live-cell imaging, due to their elastic light scattering and absorption. However, there is one special tissue that is exquisitely transparent, with minimal light scattering, termed as “biological glass”: the ocular lens. The lens acts as an optical component that allows the passage of light to the retina with minimal scatter, distortion, or absorption [7,8]. The adult lens is isolated from other tissues, has neither neural innervations nor a vascular system, and receives all required nutrients from the aqueous and vitreous humors [9]. These characteristics make the lens an ideal specimen for long-term organ culture and high-resolution, whole-organ imaging.

When observed with phase contrast optical accessories, the lens is regarded not only as a specimen for observation, but also as part of the optical system. The light from the phase condenser converges when passing through the ocular lens; however, the distortion or aberration introduced by the lens is within the acceptable range. By adjusting the condenser annulus to this new illumination state, we are able to visualize clear imaging of the curving surface of the lens. By combining the classical lens organ-culture techniques with the common inverted phase contrast microscopy, and making a small adjustment, we are able to observe lens cells with clarity comparable to single-layer cell imaging and special morphology that is totally different from the cultured lens cells. In 1968, Gierthy et al. [10] reported a similar method to observe frog lens epithelial cells in the living state; however, the microscopy technique has not been applied widely in lens cell studies.

Herein, we present some unique dynamics of lens epithelial cells (LECs) and superficial fiber cells, using our method. The images of mitosis of the LECs are similar to those previously reported [10]. The processes of apoptosis of the LECs and the dynamics of posterior fiber cells following ultraviolet A (UVA) irradiation, revealed by our method, have never been visualized before. This method is an easy to perform and rapidly executed imaging technique that can be readily applied to studies of lens physiology and pathology.

## Methods

### Chemicals

A Chromatin Condensation/Membrane Permeability/Dead Cell Apoptosis Kit with Hoechst 33342/YO-PRO®-1/propidium iodide (PI), M199 medium, and antibiotics were obtained from Invitrogen (Burlington, ON, Canada); BSA was purchased from Sigma (St. Louis, MO); phosphate buffered saline (PBS) was obtained from Hyclone (Logan, UT); and insulin-like growth factor-I (IGF-I) was obtained from PeproTech (Paris, France).

### Experimental animals

Female Wistar rats, weighing approximately 200–250 g, were obtained from the Shanghai Experimental Animal Center, Chinese Academy of Sciences (Shanghai, China). All experimental procedures conformed to the ARVO statement for the Use of Animals in Ophthalmic and Vision research and approved by an internal review committee.

### Lens organ culture

The Wistar rats were euthanized by CO_2_ inhalation. The eyeballs were surgically removed immediately after death and bathed in 75% ethyl alcohol for five seconds, and then washed with pre-warmed, sterile PBS (1×). The eyeballs were dissected under the microscope, and the lenses were placed in M199 medium and then washed twice with the same medium to remove vitreous and retinal tissue. The M199 medium was supplemented with 0.1% BSA, 100 IU/ml penicillin, and 100 μg/ml streptomycin, and it had been previously equilibrated with 5% CO_2_ for 3 h. The extracted lenses were placed in 24-well cluster plates containing 1.5–2 ml medium in each well and cultured at 37 °C, in 5% CO_2_/air. After 3 h, the lenses were observed, using our method; the lenses that were damaged during dissection were discarded. The M199 medium was renewed every day. The intact lenses stayed transparent for more than seven days.

### Phase contrast microscopy

**Figure 1:**
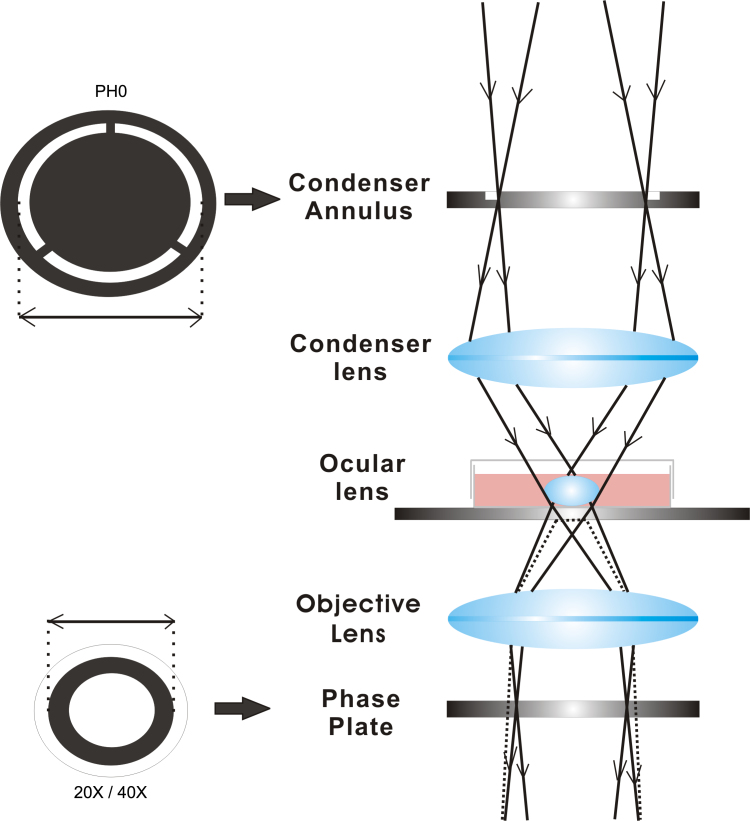
Principle of imaging of the ocular lens. The light from the phase condenser converges when passing through the ocular lens. The former corresponding condenser annulus becomes unsuitable; therefore, we used a larger condenser annulus, Ph0, to match the 20× or 40× objective lens (Leica DMI3000 B) to obtain clear images of the downward-facing surface of the lens. The diameter of the Ph0 is lager than the diameter of the phase plate on the 20× or 40× objective lens.

The lenses were observed with a Leica DMI3000 B inverted phase contrast microscope (Leica DMI3000 B; Leica, Solms, Germany). We positioned the 24-well cluster plates under the inverted phase contrast microscope and used a 5× objective lens and the corresponding Ph0 condenser annulus to focus on the downward-facing surface of the lens. Then, we switched to a 20× or 40× objective lens, but kept the Ph0 condenser annulus. The diameter of the Ph0 is lager than the diameter of the phase plate on the 20× or 40× objective lens. Because light converges when passing through the ocular lens, as shown in Figure 1, we used a larger condenser annulus, Ph0, to match the 20× or 40× objective lens (Leica DMI3000 B) and obtain clear images of the facing-down surface of the lens.

### Incubation with IGF-I

The rat lenses were incubated in the presence of IGF-I (final concentration: 20 ng/ml) for 15 h, and then they were observed for mitosis, using a phase contrast microscope.

### UVA exposure

We used a 40× objective lens and Ph0 condenser annulus to view the LECs, by focusing on a spot on the lens anterior surface. Then, we turned off the illumination, turned on the mercury lamp, and switched to an A4 excitation filter (340–380 nm). We focused 340–380 nm UVA rays on the same spot (diameter: 800 μm), and we used the fluorescence illumination of the microscope to irradiate the epithelial cells for 10 min. The fiber cells located in the posterior center were irradiated for 10 min in the same manner.

### Fluorescence Staining

The lenses were stained with a Chromatin Condensation/Membrane Permeability/Dead Cell Apoptosis Kit, which provides a rapid and convenient assay for apoptosis. It contains ready-to-use solutions of three nucleic acid stains–YO-PRO^®^-1 dye, propidium iodide, and Hoechst 33342. Green-fluorescent YO-PRO^®^-1 dye can enter apoptotic cells, whereas red-fluorescent propidium iodide (PI) cannot. Thus, after staining with YO-PRO^®^-1 dye and PI, apoptotic cells show green fluorescence, and dead cells show primarily red fluorescence and some green fluorescence. Blue-fluorescent Hoechst 33342 brightly stains the condensed chromatin of apoptotic cells and more dimly stains the normal chromatin of live cells. After UVA exposure, the rat lenses were incubated in the kit (8.1 μM Hoechst 33342, 0.1 μM YO-PRO^®^-1, and 1.5 μM PI) for 30 min at 37 °C. After the incubation period, the stained LECs were observed with a phase contrast microscope. Approximate fluorescence excitation/emission maxima: YO-PRO^®^-1: 491/509 in nm; Hoechst 33342: 350/461 in nm, bound to DNA; PI: 535/617 in nm, bound to DNA.

## Results

### Phase contrast microscopy of lens cells

**Figure 2:**
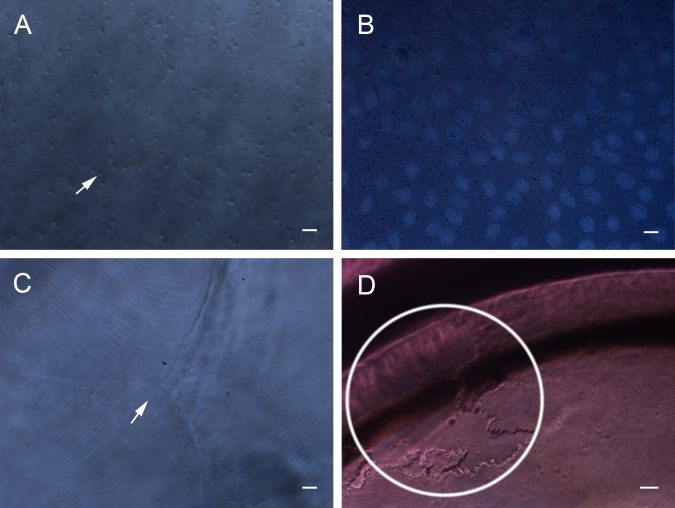
Images of the anterior and posterior surfaces of the lens. Small, rounded humps (arrow) were present all over the anterior surface, which was the major morphological feature of the LECs using our method (**A**). The chromatin of the LECs was stained with Hoechst 33342 (**B**). There were no humps on the posterior surface of the lens. The Y-suture (arrow) and the superficial fiber cells were clearly revealed (**C**). After two hours out of the incubator, the lens epithelial layer exhibited some regional changes near the equator (**D**, ring). Scale bars: **A**-**C**=10 μm; **D**=50 μm.

The intact rat lenses (without any treatment) were observed with a Leica DMI3000 B inverted phase contrast microscope. The images were vague when using the conventional method, because light converged when passing through the ocular lens. The corresponding condenser annulus became unsuitable; therefore, we used a larger condenser annulus, Ph0, to match the 20× or 40× objective lens to obtain clear images of the downward-facing surface. The diameter of the Ph0 is larger than the diameter of the phase plate on the 20× or 40× objective lens. As shown in Figure 2A-C, it was easy to distinguish the anterior and posterior surfaces of the lens. Small, round humps (arrow) were present all over the anterior surface, which was the major morphological feature of the LECs using our method (Figure 2A). The chromatin of the LECs was stained with Hoechst 33342 (Figure 2B). The LECs’ borders were not distinguishable under the microscope (Figure 2A,B), probably because of the cell–cell junctions, which served to minimize light scatter [8,11,12]. There were no humps on the posterior surface of the lens. As shown in Figure 2C, the Y-suture and the superficial fiber cells were clearly revealed by our method. During our observation, we noticed an interesting phenomenon. After 2h out of the incubator, the lens epithelial layer exhibited some regional changes near the equator (Figure 2D, ring). This phenomenon disappeared quickly after renewing the medium, equilibrated with 5% CO_2_ at 37 °C. It is suggested that slight changes of the environmental conditions, such as medium pH and temperature, might induce morphological changes of the LECs; therefore, the lens should be kept under proper conditions during observation.

### Mitosis of the LECs

**Figure 3:**
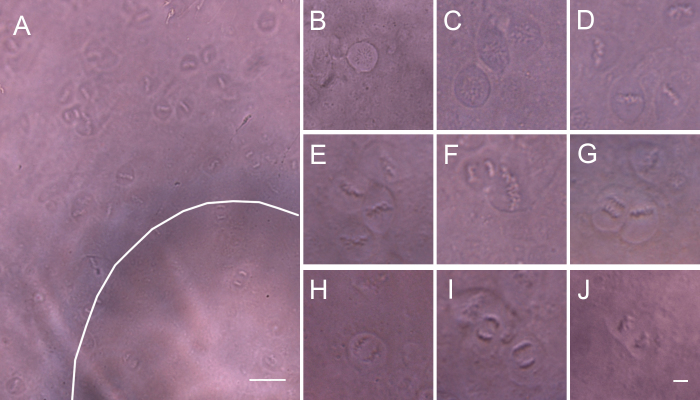
Imaging of mitosis of the LECs. Numerous mitotic figures were present all over the anterior surface after 15 h of incubation with 20 ng/ml IGF-I (**A**). The chromosomes and the profiles of the cell bodies were distinctly visible, and dividing cells in various stages could easily be observed (**B**–**J**). A vague, dark ring (arc) was located in the center of the lens surface (**A**). The curved surface of the lens permitted visualization of a small area of cells in the focal plane. Scale bars **A**=50 μm; **B**–**J**=10 μm.

It is known that cell division occurs exclusively in the anterior epithelium, primarily in an annular zone (the “proliferative” or “germinative” zone) underlying the ciliary body [13,14]. However, in our experiment, numerous mitotic figures were present all over the anterior surface after 15 h of incubation with 20 ng/ml IGF-I (Figure 3A). IGF-I is an important growth factor in the ocular fluid, which can strongly stimulate DNA synthesis and cell division [15,16]. The video captured the entire LEC mitosis process. The chromosomes and the profiles of the cell bodies were distinctly visible when the LECs were dividing. Newly formed daughter cells rapidly merged into the background epithelium and became invisible. Dividing cells in various stages could be easily observed through the same lens (Figure 3). The vague, dark ring located in the center of the lens surface was not caused by the opacities of the lens, but by the residual mismatching of the optical component (Figure 3A, arc). The curved surface of the lens permitted visualization of a small area of cells in the focal plane. The number, location, and stages of the dividing cells were readily observed (Figure 3), and the duration of each phase of the mitotic cycle could be directly calculated (video).

### Apoptosis of the LECs

**Figure 4:**
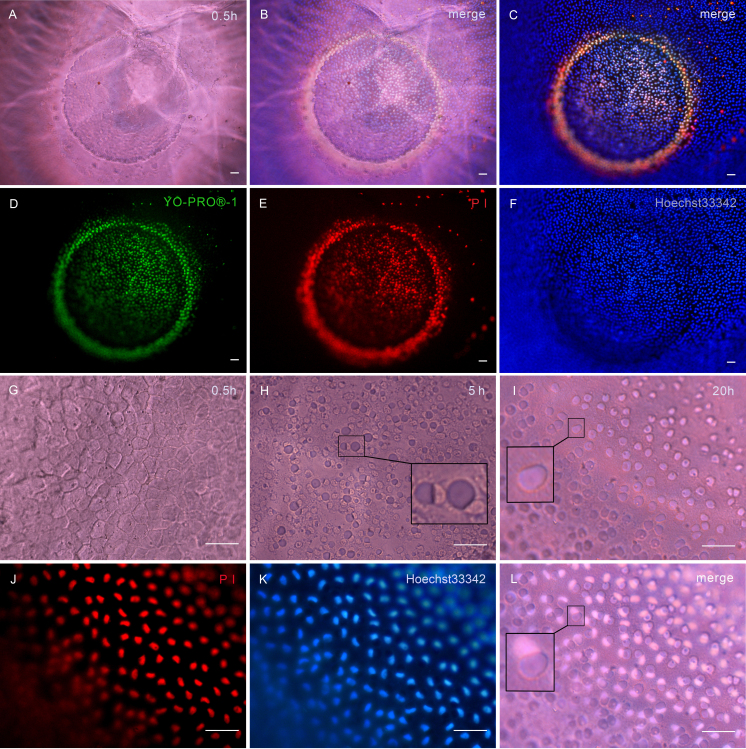
Imaging of apoptosis of the LECs. The round apoptosis area could be readily distinguished after 30 min (**A**–**C**). Apoptotic cells showed green fluorescence (YO-PRO^®^-1; **D**), and dead cells showed primarily red fluorescence (PI; **E**, **J**). Blue-fluorescent Hoechst 33342 stained the condensed chromatin of apoptotic cells and the normal chromatin of live cells (**F**, **K**). **C**: A merging of **D**, **E**, and **F**. **B**: A merging of **A** and **C**. After 30 min, the cell borders and small, rounded humps were clearly observed (**G**). After five h, the nuclei of the apoptotic cells moved aside, and round caves were left in the original sites (**H**). After 20 h, the nuclei were invisible under an optical microscope, and there were only round caves in the irradiation area (**I**). Fluorescent staining showed that all of the cells were dead, and the nuclei lay adjacent to the round caves (**J**–**L**). **L**: A merging of **I**, **J**, and **K**. High-magnification images of the apoptotic cells are shown in the rectangles. Scale bars: **A**–**L**=50 μm.

Typical apoptotic morphology of LECs after UV exposure includes chromatin condensation, nuclear fragments of varying size, a clustering of apoptotic bodies, cell shrinkage, and budding [17,18]. Morphological dynamics of UV-induced apoptosis of LECs in their physiologic location had never been visualized before. In our experiments, we applied a new irradiation method to induce apoptosis of the LECs. We used the incident ultraviolet light of the microscope to irradiate a small area of epithelial (diameter: 800 μm) and, after exposure to UVA rays (340–380 nm) for 10 min, the lens was observed by our method. As shown in Figure 4A-C, the round apoptosis area could be readily distinguished after 30 min. Apoptotic cells showed green fluorescence (YO-PRO®-1; Figure 4D), and dead cells showed primarily red fluorescence (PI; Figure 4E,J). Blue-fluorescent Hoechst 33342 brightly stained the condensed chromatin of the apoptotic cells and more dimly stained the normal chromatin of the live cells (Figure 4F,K). The normally invisible LECs became obviously distinguishable when they were dead. The cell borders and the small, rounded humps mentioned previously could be observed clearly (Figure 4G). After five h, the apoptotic cells showed a unique phenomenon, which, we believe, has never been seen before. The nuclei of the apoptotic cells moved aside, and round caves were left in the original sites (Figure 4H, rectangle). After 20 h, the nuclei were invisible under an optical microscope, and there were only round caves in the irradiation area (Figure 4I, rectangle). However, fluorescent staining showed that all of the LECs were dead, and the nuclei lay adjacent to the round caves (Figure 4L, rectangle). In their living state, LECs are confined to a limited space, making the dynamics of cell apoptosis quite different from the cultured cells. Our method provides a better way to record the morphological changes of apoptosis in LECs.

### Dynamics of the posterior fiber cells following UVA irradiation

**Figure 5:**
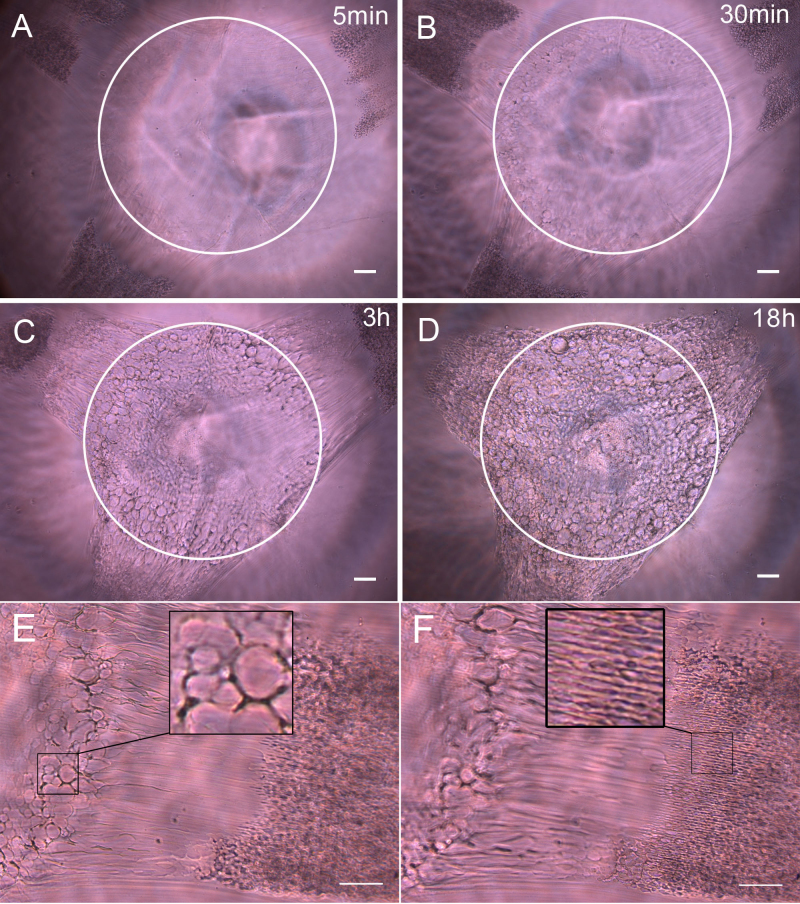
Imaging of the morphological dynamics of posterior fiber cells following UVA irradiation. The morphological alterations of the fibers after 5 min (**A**), 30 min (**B**), 3 h (**C**), and 18 h (**D**) are shown. The round irradiation area (ring) exhibited no obvious changes after 5 min. The fibers on this area aggregated into globules after 30 min (**B**, **C**). After 18 h, the aggregation area enlarged to a triquetrous opaque and showed a liquid tremor (**D**). Three plaques of damage appeared on the adjacent area after 5 min (**A**), which became more severe after 30 min (**B**). This damage gradually faded, and it completely disappeared after 18 h (**C**, **D**). High-magnification images of the fiber changes after 30 min are shown in **E** and **F**. There are two different types of damages: the fibers on the irradiation area aggregated into globules (**E**, rectangle); the extracellular space between the fibers on the adjacent area became enlarged, but the fibers themselves did not break down (**F**, rectangle). Scale bars: **A**–**F**=50 μm.

Cataract formation is related to oxidative stress induced by continued intraocular penetration of light and consequent photochemical generation of reactive oxygen species [19-21]. During this process, lenses may show incomplete denucleation, enlargement of extracellular space, disarrangement of cell packing, high molecular aggregation, formation of small intercellular vacuoles, etc [8,22]. We irradiated the superficial fiber cells in the posterior center of the lens for ten minutes, using the same method described previously. The morphological alterations of the fibers after 5 min, 30 min, 3 h, and 18 h are shown in Figure 5A–D. The round irradiation area (ring) exhibited no obvious changes after five minutes (Figure 5A). The fibers on this area aggregated into globules after 30 min (Figure 5B and C). After 18 h, the aggregation area enlarged to a triquetrous opaque and showed a liquid tremor (Figure 5D). The damage caused by the UVA irradiation seemed to transmit along the fiber to the adjacent area. Three plaques of damage appeared on the adjacent area after 5 min, which became more severe after 30 min (Figure 5A,B). This damage gradually faded, and it completely disappeared after 18 h (Figure 5C,D). High-magnification images of the fiber changes after 30 min are shown in Figure 5E,F. The fibers on the irradiation area aggregated into globules (Figure 5E, rectangle); however, the fibers on the adjacent area showed different morphological changes. The extracellular space between fibers became enlarged, but the fibers themselves did not break down (Figure 5F, rectangle). It is indicated that there are two different types of damages—one is caused by protein aggregation, and the other is caused by the destruction of the cell junctions. The latter, we believe, is reversible.

## Discussion

To some degree, the optical performance of the living lens exceeds that of a simple glass lens [8]. Our method takes advantage of the optical properties of the ocular lens, regarding it as part of the microscope’s optical system, which requires the lens to be extremely transparent. A slight loss of transparency of the lens can result in dim or blurred images, which might be the biggest limitation of our method. However, it provides a highly sensitive detection method for morphological changes during the early stages of cataract formation in vitro. Compared to grid- and dark-field illumination photography [23], our approach can provide more subtle information about the lens.

Our method offers a new angle for studying LECs. Using this method, we can easily observe the morphological changes of LECs in their natural state. Unlike cultured cell studies, organ-culture studies can provide more spatial messages and more information about cell–cell and cell–organ interaction. We can grasp the features of LECs by visualizing them in different positions. Without using stain, we can determine the number, location, and stages of the mitotic LECs. Thus, this method could be a powerful tool for studying lens stem cells. In cultured cell studies, dead LECs detach from the dish, making it impossible to record the complete process of apoptosis. With our method, however, as the whole lens is encapsulated, the dead LECs stay in their anatomic positions, and every step of LEC apoptosis can be recorded. In our experiments, the incident ultraviolet light of the microscope was used to induce apoptosis of the LECs. The advantages of this new irradiation method are that the irradiation location can be precisely controlled; the damage is limited, and it will not affect the transparency of the lens. We hope our method will facilitate study of the lens as a spatial, functional, universally associated organ.

Fiber cells are the main elements in all vertebrate lenses, comprising more than 95% of tissue volume [8], and most cataracts occur specifically in the fibers [22]. However, because it is difficult to culture lens fiber cells in vitro, the morphological dynamics of lens fibers during cataract formation have been observed rarely. Our microscopy method, however, makes lens fibers suitable specimens for study, as we can directly observe the morphological changes of the superficial lens fibers. We have recorded two types of damage to fiber cells following UVA radiation. Previous studies have shown that the close apposition of lens fibers can minimize the scattering at the cell borders, and protein aggregation is an important cause of cataracts [8,22,24]. Our findings further certify these theories.

In this article, we present a high-resolution detection method for cell morphological changes within the whole lens. Combined with live-cell fluorescence staining techniques [25], we can study the cellular organelle and protein changes in LECs and fibers. We believe this method can open a new window for studying lens physiology and pathology.

## Supplementary Material

video

## References

[1] Taylor VL, Al-Ghoul KJ, Lane CW, Davis VA, Kuszak JR, Costello MJ (1996). Morphology of the normal human lens.. Invest Ophthalmol Vis Sci.

[2] Schmolze DB, Standley C, Fogarty KE, Fischer AH (2011). Advances in microscopy techniques.. Arch Pathol Lab Med.

[3] Bantseev VL, Herbert KL, Trevithick JR, Sivak JG (1999). Mitochondria of rat lenses: distribution near and at the sutures.. Curr Eye Res.

[4] Ji N, Shroff H, Zhong H, Betzig E (2008). Advances in the speed and resolution of light microscopy.. Curr Opin Neurobiol.

[5] Verschueren H (1985). Interference reflection microscopy in cell biology: methodology and applications.. J Cell Sci.

[6] Unwin PN (1971). Phase contrast and interference microscopy with the electron microscope.. Philos Trans R Soc Lond B Biol Sci.

[7] Delaye M, Tardieu A (1983). Short-range order of crystallin proteins accounts for eye lens transparency.. Nature.

[8] Bassnett S, Shi Y, Vrensen GF (2011). Biological glass: structural determinants of eye lens transparency.. Philos Trans R Soc Lond B Biol Sci.

[9] Wormstone IM, Wride MA (2011). The ocular lens: a classic model for development, physiology and disease.. Philos Trans R Soc Lond B Biol Sci.

[10] Gierthy JF Borrow SN Rothstein H Microscopy of living epithelial cells upon the intact ocular lens in culture. Exp Cell Res 1968 50 476 9 4925966

[11] Sugiyama Y, Prescott AR, Tholozan F, Ohno S, Quinlan RA (2008). Expression and localisation of apical junctional complex proteins in lens epithelial cells.. Exp Eye Res.

[12] Lo WK, Harding CV (1983). Tight junctions in the lens epithelia of human and frog: freeze-fracture and protein tracer studies.. Invest Ophthalmol Vis Sci.

[13] McAvoy JW (1978). Cell division, cell elongation and distribution of alpha-, beta- and gamma-crystallins in the rat lens.. J Embryol Exp Morphol.

[14] Griep AE (2006). Cell cycle regulation in the developing lens.. Semin Cell Dev Biol.

[15] Arnold DR, Moshayedi P, Schoen TJ, Jones BE, Chader GJ, Waldbillig RJ (1993). Distribution of IGF-I and -II, IGF binding proteins (IGFBPs) and IGFBP mRNA in ocular fluids and tissues: potential sites of synthesis of IGFBPs in aqueous and vitreous.. Exp Eye Res.

[16] Reddan JR, Wilson-Dziedzic D (1983). Insulin growth factor and epidermal growth factor trigger mitosis in lenses cultured in a serum-free medium.. Invest Ophthalmol Vis Sci.

[17] Shui YB, Sasaki H, Pan JH, Hata I, Kojima M, Yamada Y, Hirai KI, Takahashia N, Sasaki K (2000). Morphological observation on cell death and phagocytosis induced by ultraviolet irradiation in a cultured human lens epithelial cell line.. Exp Eye Res.

[18] Michael R, Vrensen GF, van Marle J, Gan L, Soderberg PG (1998). Apoptosis in the rat lens after in vivo threshold dose ultraviolet irradiation.. Invest Ophthalmol Vis Sci.

[19] Varma SD, Kovtun S, Hegde KR (2011). Role of ultraviolet irradiation and oxidative stress in cataract formation-medical prevention by nutritional antioxidants and metabolic agonists.. Eye Contact Lens.

[20] Mafia K, Gupta R, Kirk M, Wilson L, Srivastava OP, Barnes S (2008). UV-A-induced structural and functional changes in human lens deamidated alphaB-crystallin.. Mol Vis.

[21] Williams DL (2006). Oxidation, antioxidants and cataract formation: a literature review.. Vet Ophthalmol.

[22] Balasubramanian D, Bansal AK, Basti S, Bhatt KS, Murthy JS, Rao CM (1993). The biology of cataract. The Hyderabad Cataract Research Group.. Indian J Ophthalmol.

[23] Meyer LM, Dong X, Wegener A, Soderberg P (2007). Light scattering in the C57BL/6 mouse lens.. Acta Ophthalmol Scand.

[24] Swamy MS, Abraham EC (1987). Lens protein composition, glycation and high molecular weight aggregation in aging rats.. Invest Ophthalmol Vis Sci.

[25] Massignani M, Canton I, Sun T, Hearnden V, Macneil S, Blanazs A, Armes SP, Lewis A, Battaglia G (2010). Enhanced fluorescence imaging of live cells by effective cytosolic delivery of probes.. PLoS ONE.

